# Supplementary material for: RNase H-based analysis of synthetic mRNA 5′ cap incorporation
Source: RNA. 2022 Aug;28(8):1144–55. doi: 10.1261/rna.079173.122 (PMC9297845; doi:10.1261/rna.079173.122)
Supplement: Supplemental Material [file supp_28_8_1144__DC1.html]

RNase H-based analysis of synthetic mRNA 5’ cap incorporation — RNase H-based analysis of synthetic mRNA 5′ cap incorporation — Supplemental Material 

# RNase H-based analysis of synthetic mRNA 5′ cap incorporation

## Supplemental Material

- Supplemental\_Figures.pptx
