# Supplementary material for: RNase H-based analysis of synthetic mRNA 5′ cap incorporation
Source: RNA. 2022 Aug;28(8):1144–55. doi: 10.1261/rna.079173.122 (PMC9297845; doi:10.1261/rna.079173.122)
Supplement: Supplemental Material [file supp_079173.122_Supplemental_Figures.pptx]

## Slide 1
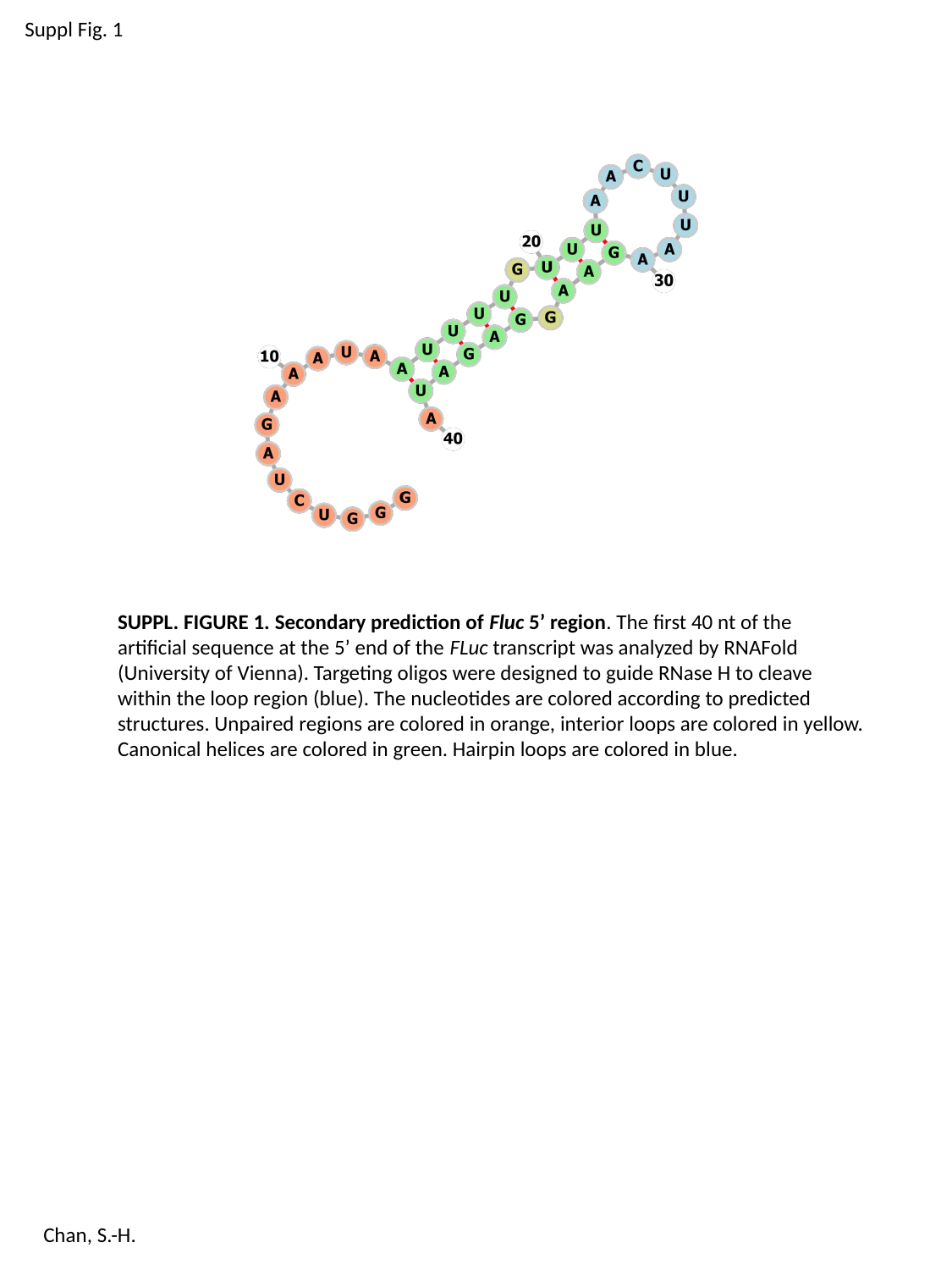

Suppl Fig. 1
SUPPL. FIGURE 1. Secondary prediction of Fluc 5’ region. The first 40 nt of the artificial sequence at the 5’ end of the FLuc transcript was analyzed by RNAFold (University of Vienna). Targeting oligos were designed to guide RNase H to cleave within the loop region (blue). The nucleotides are colored according to predicted structures. Unpaired regions are colored in orange, interior loops are colored in yellow. Canonical helices are colored in green. Hairpin loops are colored in blue.
Chan, S.-H.

## Slide 2
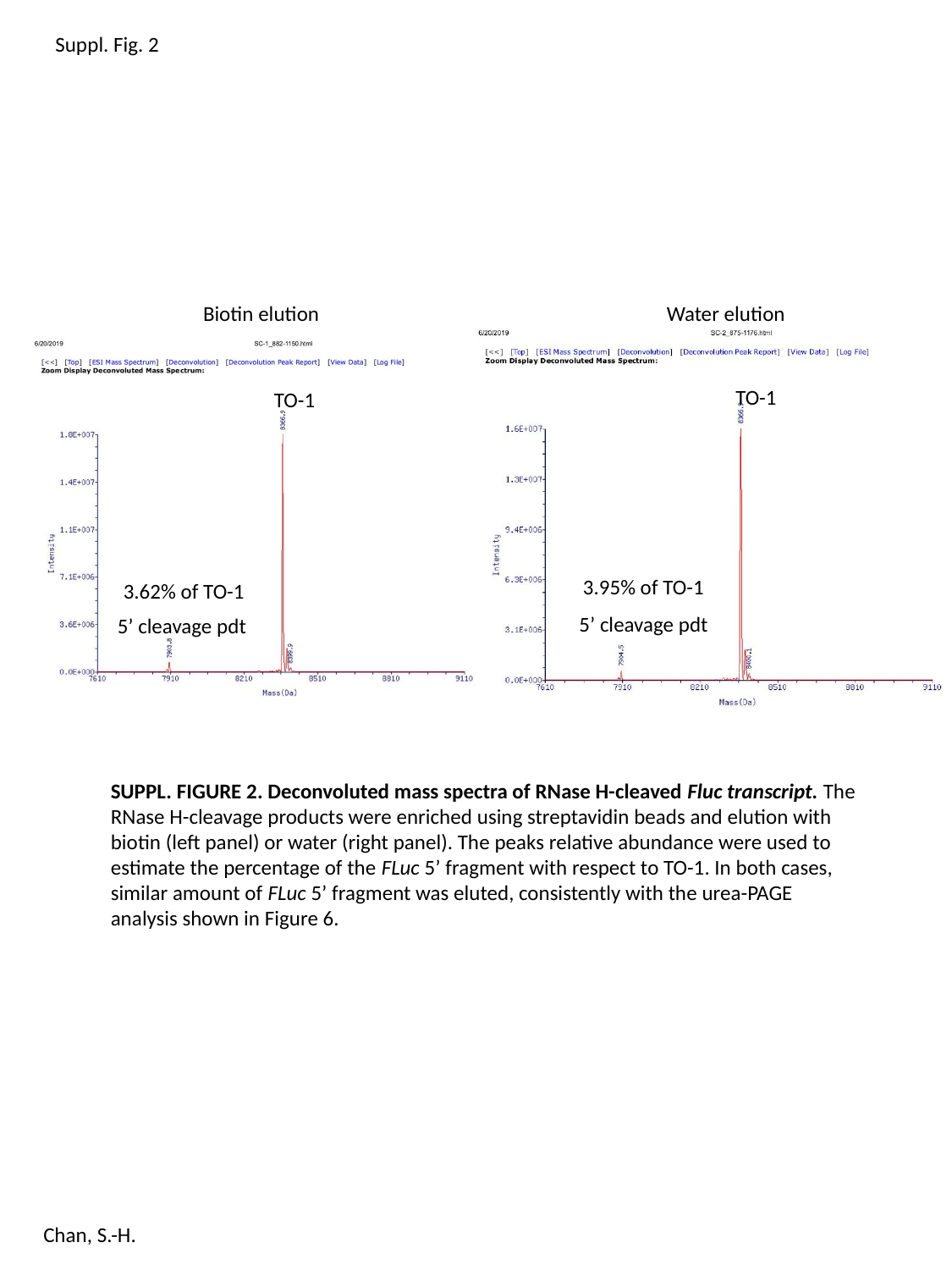

Suppl. Fig. 2
Biotin elution
TO-1
5’ cleavage pdt
Water elution
TO-1
5’ cleavage pdt
3.95% of TO-1
3.62% of TO-1
SUPPL. FIGURE 2. Deconvoluted mass spectra of RNase H-cleaved Fluc transcript. The RNase H-cleavage products were enriched using streptavidin beads and elution with biotin (left panel) or water (right panel). The peaks relative abundance were used to estimate the percentage of the FLuc 5’ fragment with respect to TO-1. In both cases, similar amount of FLuc 5’ fragment was eluted, consistently with the urea-PAGE analysis shown in Figure 6.
Chan, S.-H.

## Slide 3
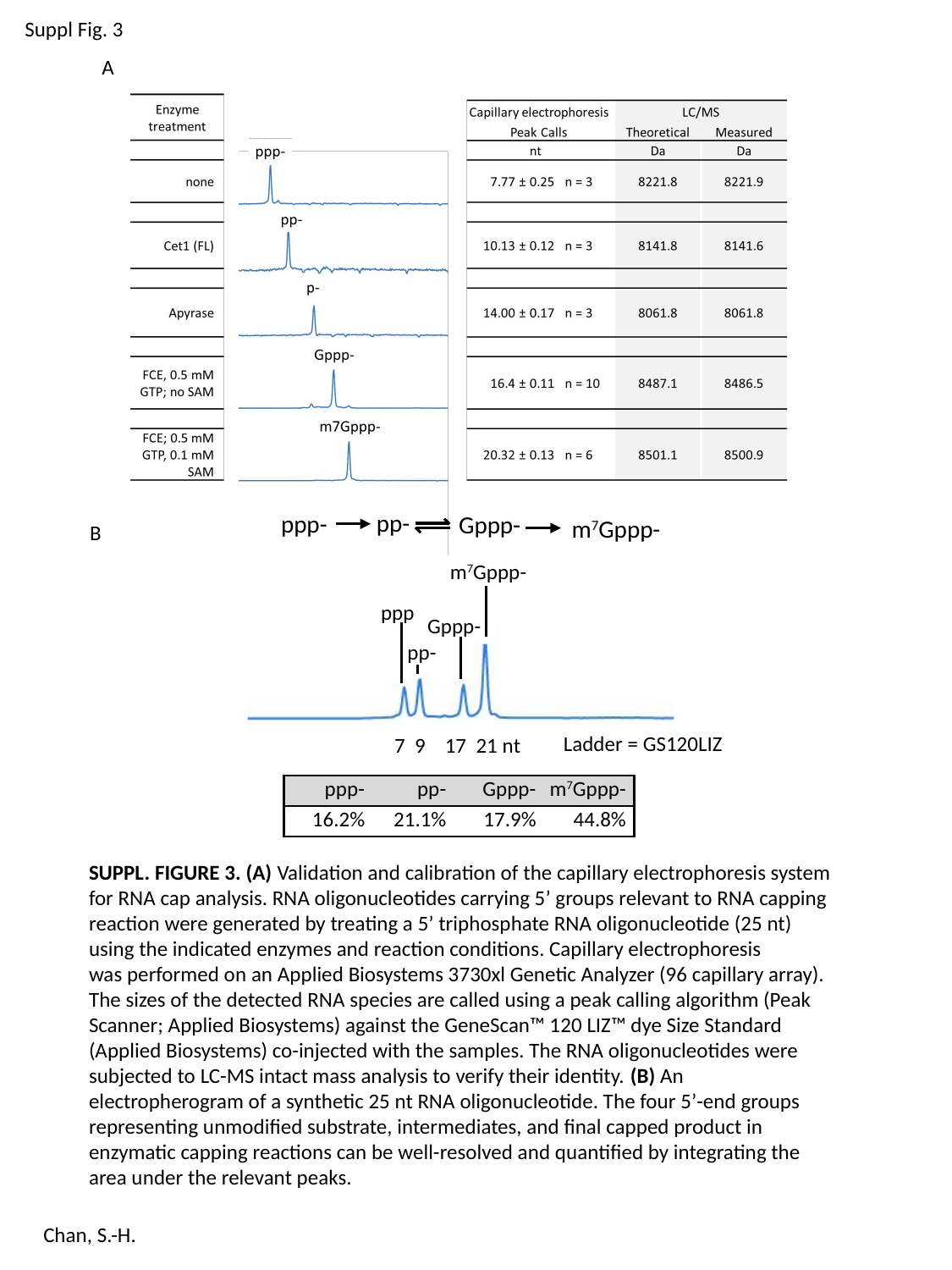

Suppl Fig. 3
A
pp-
ppp-
Gppp-
m7Gppp-
B
m7Gppp-
ppp-
Gppp-
pp-
20180413_H3C2_SAM_low_conc_temp
Sample #8
Ladder = GS120LIZ
7 9 17 21 nt
| ppp- | pp- | Gppp- | m7Gppp- | |
| --- | --- | --- | --- | --- |
| 16.2% | 21.1% | 17.9% | 44.8% | |
SUPPL. FIGURE 3. (A) Validation and calibration of the capillary electrophoresis system for RNA cap analysis. RNA oligonucleotides carrying 5’ groups relevant to RNA capping reaction were generated by treating a 5’ triphosphate RNA oligonucleotide (25 nt) using the indicated enzymes and reaction conditions. Capillary electrophoresis was performed on an Applied Biosystems 3730xl Genetic Analyzer (96 capillary array). The sizes of the detected RNA species are called using a peak calling algorithm (Peak Scanner; Applied Biosystems) against the GeneScan™ 120 LIZ™ dye Size Standard (Applied Biosystems) co-injected with the samples. The RNA oligonucleotides were subjected to LC-MS intact mass analysis to verify their identity. (B) An electropherogram of a synthetic 25 nt RNA oligonucleotide. The four 5’-end groups representing unmodified substrate, intermediates, and final capped product in enzymatic capping reactions can be well-resolved and quantified by integrating the area under the relevant peaks.
Chan, S.-H.

## Slide 4
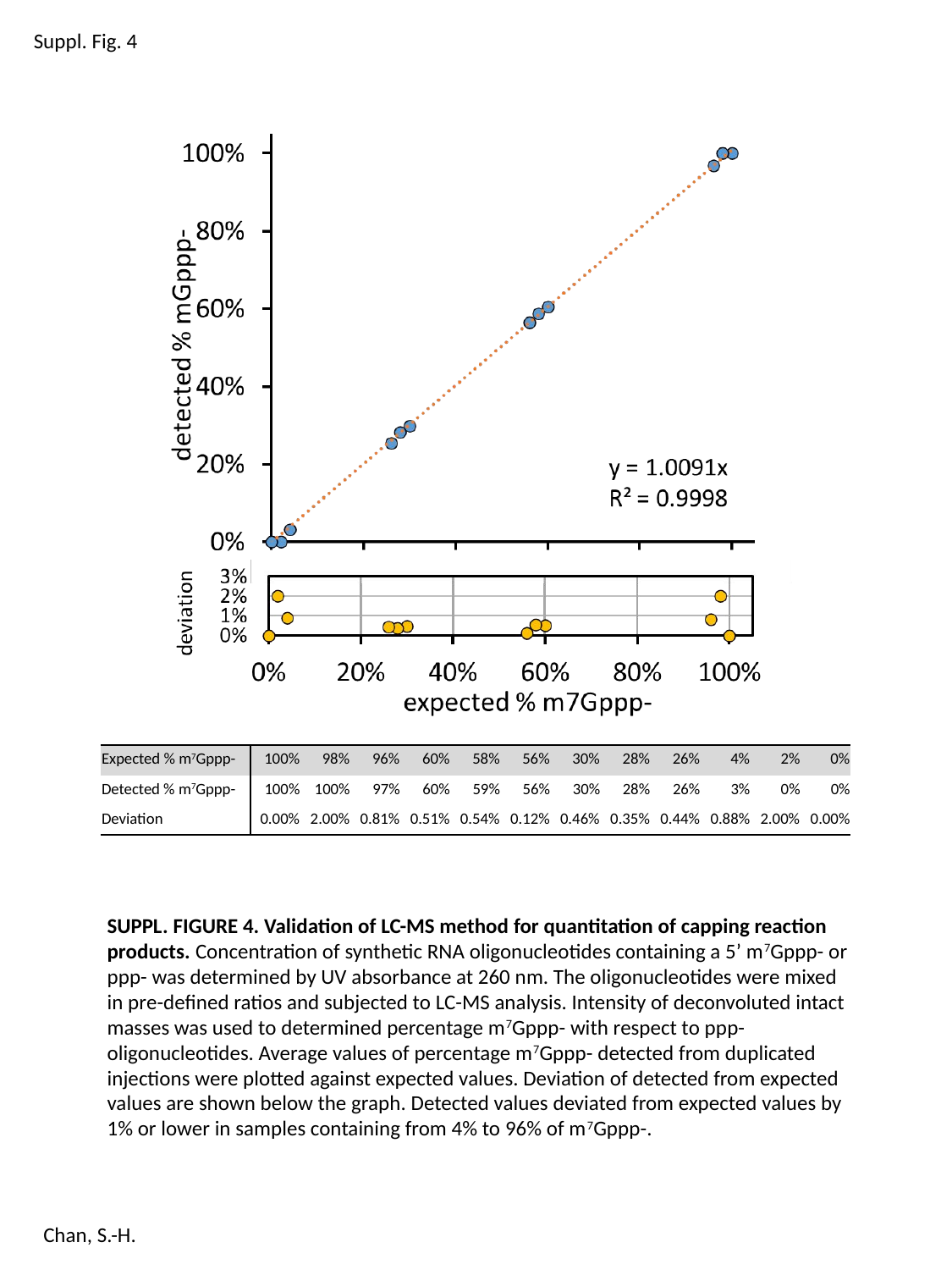

Suppl. Fig. 4
| Expected % m7Gppp- | 100% | 98% | 96% | 60% | 58% | 56% | 30% | 28% | 26% | 4% | 2% | 0% |
| --- | --- | --- | --- | --- | --- | --- | --- | --- | --- | --- | --- | --- |
| Detected % m7Gppp- | 100% | 100% | 97% | 60% | 59% | 56% | 30% | 28% | 26% | 3% | 0% | 0% |
| Deviation | 0.00% | 2.00% | 0.81% | 0.51% | 0.54% | 0.12% | 0.46% | 0.35% | 0.44% | 0.88% | 2.00% | 0.00% |
SUPPL. FIGURE 4. Validation of LC-MS method for quantitation of capping reaction products. Concentration of synthetic RNA oligonucleotides containing a 5’ m7Gppp- or ppp- was determined by UV absorbance at 260 nm. The oligonucleotides were mixed in pre-defined ratios and subjected to LC-MS analysis. Intensity of deconvoluted intact masses was used to determined percentage m7Gppp- with respect to ppp-oligonucleotides. Average values of percentage m7Gppp- detected from duplicated injections were plotted against expected values. Deviation of detected from expected values are shown below the graph. Detected values deviated from expected values by 1% or lower in samples containing from 4% to 96% of m7Gppp-.
Chan, S.-H.

## Slide 5
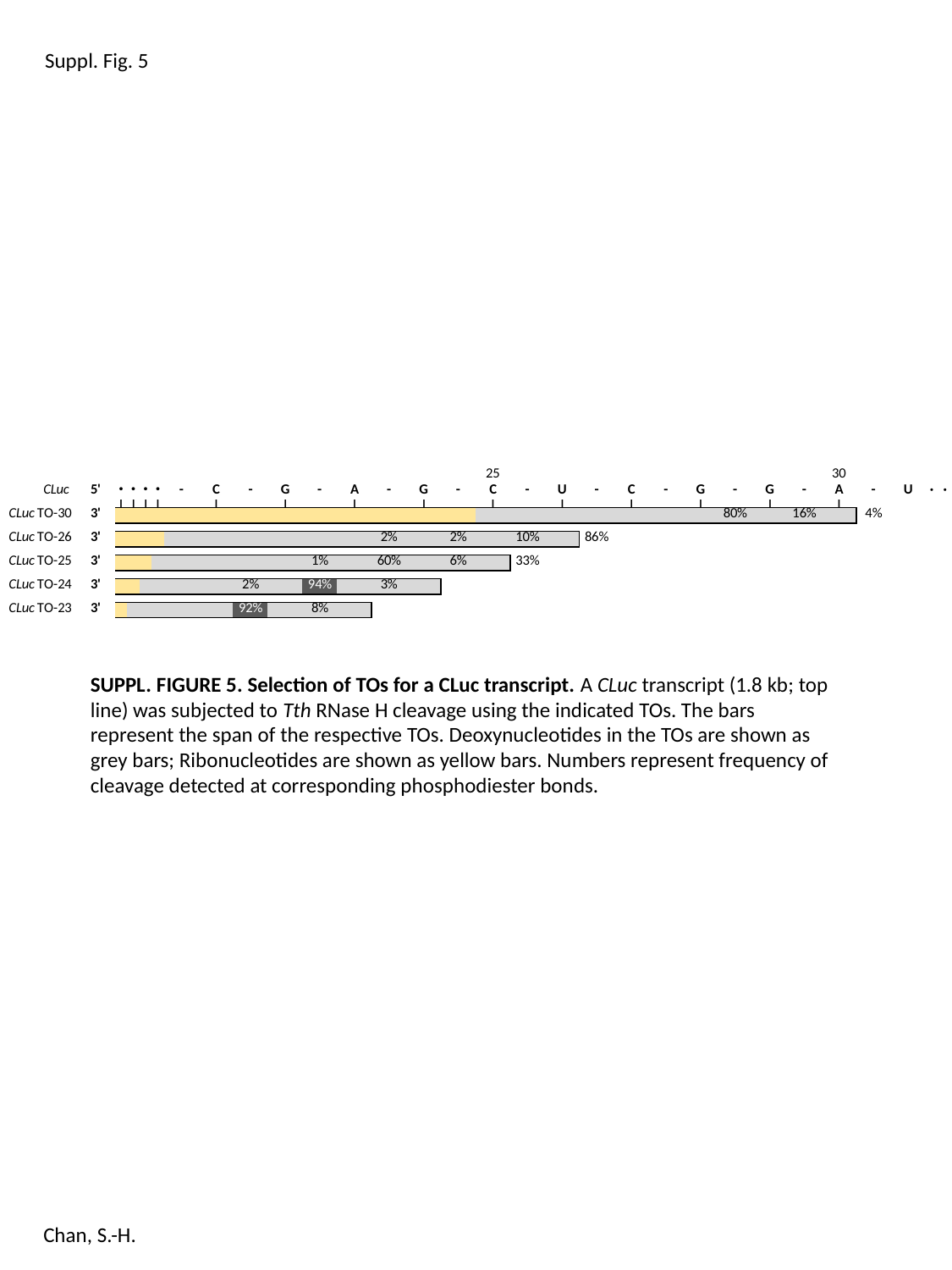

Suppl. Fig. 5
SUPPL. FIGURE 5. Selection of TOs for a CLuc transcript. A CLuc transcript (1.8 kb; top line) was subjected to Tth RNase H cleavage using the indicated TOs. The bars represent the span of the respective TOs. Deoxynucleotides in the TOs are shown as grey bars; Ribonucleotides are shown as yellow bars. Numbers represent frequency of cleavage detected at corresponding phosphodiester bonds.
Chan, S.-H.
